# Smartphone applications for sleep tracking: rating and perceptions about behavioral change among users

**DOI:** 10.5935/1984-0063.20210007

**Published:** 2022

**Authors:** Reema A. Karasneh, Sayer I. Al-Azzam, Karem H. Alzoubi, Sahar Hawamdeh, Anan S. Jarab, Mohammad B. Nusair

**Affiliations:** 1 Yarmouk University, Department of Basic Medical Sciences - Irbid - Jordan.; 2 Jordan University of Science and Technology, Department of Clinical Pharmacy - Irbid - Jordan.; 3 University of Sharjah, Department of Pharmacy Practice and Pharmcotherapeutics - Sharjah - UAE.; 4 Yarmouk University, Department of Pharmacy Practice - Irbid - Jordan.

**Keywords:** Sleep Tracker, Sleep Health, Smartphone Applications, Behavioural Change, Consumer Sleep Technology, Mobile Devices

## Abstract

**Introduction:**

This study aims to assess existing sleep apps for mobile phones to determine the perceived effect of these applications on user’s attitudes, knowledge, willingness to change, and its likelihood to change behavior from a user’s perspective.

**Material and Methods:**

A systematic search was conducted through Google play store and iTunes Apple store using terms related to sleep tracking. Apps were evaluated using Mobile Application Rating Scale (MARS) tool for assessing and classifying mobile health applications quality. Additionally, a convenience sample of subjects were asked to evaluate the included apps for perceived sleep behavior changes.

**Results:**

The average MARS app quality score on a 5-point scale was 3.3. Between 30-50% of participants believed that sleep tracker apps are likely to increase awareness about sleep patterns and sleep hygiene, infuence sleep hygiene habits, and are likely to encourage help seeking for sleep hygiene when required.

**Conclusion:**

Apps available for sleep self-management and tracking may be valuable tools for self-management of sleep disorder and/or improving sleep quality, yet they require improvement in terms of quality and content, highlighting the need for further validity studies.

## INTRODUCTION

Sleep occupies between 20% and 40% of the human day. Even prehistoric evidence suggests the importance of sleep in human life, which is consistent with archaeological and historical accounts of sleep having a prominent and important role even in early human societies^1,2^. Therefore, sleep is considered a vital component of human life and essential for a person’s health and well-being. It plays a critical role in different brain functions, such as facilitating memory consolidation, impacting cognitive and neurobehavioral performance, mood regulation, and subserving the brain clearance of various toxic waste products^3,4,5,6,7,8,9^.

According to the National Sleep Foundation, young adults aged between 18-25 years and adults aged 26 to 64 years should optimally have 7-9 hours of sleep. On the other hand, adults aged 65 years or older should sleep for 7-8 hours daily, not exceeding 9 hours and not getting less than 5 hours of sleep^2^. Sleep problems are widely prevalent, including deficiency in sleep quantity and quality and disturbance of sleep continuity, collectively referred to as sleep disruption. The characteristics of normal healthy sleep are a sufficient duration with regular and appropriate timing, and good quality in the absence of disorders and disturbances related to sleep^2^. On the long-term, sleep disturbances not only cause poor activity, fatigue, and decreased cognitive performance, but also increase the risk of early mortality, and several comorbid conditions such as cardiovascular disease, diabetes, hypertension, obesity, cancer, and depression^10,11,12,13,14,15,16,17,18^. Thus, getting enough sleep with good quality is important for human health and well-being.

Electronic health (eHealth) literacy is defined as “the ability of people to use emerging information and communications technologies to improve or enable health and health care”^19^. Mobile devices have become an inspirational and integral part of modern living, by being typically customized to the specific needs of individuals, creating deep personal relationships with their users^20^. Patients, as consumers, have a great need for mobile health (mHealth) applications that can help them with their medication adherence, as suggested by the 106% increase of iOS mHealth apps available to the public from 2013 to 2015. Interestingly, the mHealth apps grew from 43,689 in 2013 to 90,088 in 2015^21^.

Self-management applications on a smartphone have been advocated as a means to help people with sleep disturbance to achieve better levels of sleep control and better sleep quality outcomes. With increased mobile phone ownership on a global scale, a rapid development in mHealth technologies allows users to self-monitor and visualize their sleep patterns, symptoms, and behavioural data and aid them in taking appropriate actions on a potentially daily basis^22^. Most of the available sleep tracking apps are accelerometer-based and can employ built-in mobile sensors such as microphones and light detectors to obtain sleep data^1^. However, and unlike accelerometry used in standard sleep assessment tools (e.g., actigraphy), mobile accelerometer operates via undefined and varying algorithms. Thus, their use remains a concern due to the lack of validation studies^1,22^.

These applications provide a wide range of functions, including smart alarms, sleep aids, sound recording during sleep, and analysis of sleep. Other applications were designed to assist healthcare professionals in monitoring and screening their patients for habitual snoring and obstructive sleep apnea^23^. A few health trackers were compared to standard sleep assessment tests including polysomnography (PSG), wrist actigraphy, and the Pittsburgh sleep quality index (PSQI)^24^. Parameters assessed in validation studies included sleep onset latency, total sleep time, snoring events, sleep stages, and sleep efficacy. Most applications have shown good correlation to wrist actigraphy but not PSG. Moreover, a drop in reliability was commonly seen in clinical populations compared to healthy users, a trend also seen with conventional actigraphy^24,25,26,27,28,29^.

Sleep health application may be valuable for user self-management and improvement of sleep hygiene. In addition, they may help increase awareness and promote help seeking regarding sleep-related issues. However, the severe lack of validation studies raises concerns around their use and limits their function as alternatives to standard clinical tools. Moreover, assessment of available applications is necessary to guide physician recommendations of sleep tracker apps for patient use. To our knowledge, this is the first study to assess the perceived behavioural changes associated with sleep health applications.

## MATERIAL AND METHODS

### Systematic search criteria

**Figure 1.**
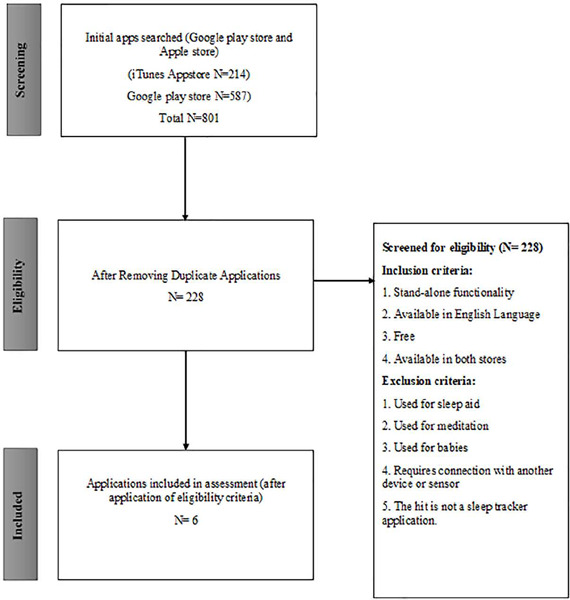
Schematic presentation of search responses processing.

Mobile apps for sleep tracking were identifed by searching the Apple iTunes Store and Android Google Play store using mobile devices. Sleep trackers were defined as applications that measure sleep data such as sleep duration and/or quality^30^. The following search terms were used: “sleep tracker”, “sleep analysis”, and “sleep cycle”, as they are the most commonly used description of these term. Each term was separately searched at both app stores during November 2019. Apps retrieved using the above-mentioned search terms were downloaded onto their respective devices (iPhone or Android) and then duplicates were removed. Search was done out of Jordan. Apps were included if they were available in both stores in English language, have a stand-alone functionality (i.e., not part of another app), and were free to download. Apps were excluded if their core function was sleep aid that include features such as calming visual graphics, relaxing music, nature sounds, and/or white noise^31^, meditation apps; if they were used for babies, if they need to be connected with another device or sensor to conduct their core function, or if they were paid apps. Apps that were found not to be a sleep-tracking app were also excluded (Figure 1).

### Data extraction

A detailed review of the included apps was conducted, and data was independently extracted by two reviewers. The data included information on classification, and objective and subjective qualities, as described in the mobile application rating scale (MARS). Classification of data included characteristics of apps such as descriptive information on rating and technical aspects such as password protection, confidentiality, and security. Objective quality included engagement, functionality, aesthetics, and information features. Presence or absence of objective quality features was assessed in extracted information and if not found then was considered absent^32,33^.

### Assessment of sleep tracker mobile apps

The MARS tool was used for assessing the quality of health mobile apps for sleep tracking. The tool included three main sections; app description, application objective section, which is divided into 4 scales assessing application engagement, functionality, aesthetics, and quality of information, and subjective application quality, which evaluated overall user satisfaction. A 5-point Likert scale was used for MARS items scoring. The final MARS score included average quality score and subjectivity scores.

### Perception about behavioural change assessment

We assessed users’ perceptions about the behavioural changes associated with the retrieved apps using an app-specific behavioural change MARS subscale^34^. The tool was developed on a web-based platform to facilitate completion and collection of data (Supplementary fle I). The protocol of this study was approved by the institutional review board of Jordan University of Science and Technology, Jordan, and written informed consent form were obtained from study participants. For each app, five independent reviews from participants from the community were considered. Therefore, invitations were sent via email using convenience-sampling technique (i.e., depending on accessible e-mail lists to the research team, which included more than 1,000 e-mails).

Participants in the study were asked to download and use one of the sleep tracker apps for two weeks and to fll-out the questionnaire that consisted of five questions related to behaviour change. Content related to the behaviors was reviewed and scored for each app to assess potential impact on user sleep hygiene awareness of the importance of addressing sleep patterns, knowledge or understanding, attitudes toward improving, intentions or motivations to change or address, help-seeking, and behavior change. Each of these questions had five-points, from one to five; where one represented strongly disagree and five represented strongly agree and average score was calculated for each app. Sleep hygiene was defined as by the criteria set by the National Sleep Foundation, USA (https://www.sleepfoundation.org/articles/sleep-hygiene).

## RESULTS

### Evaluation of sleep tracker apps

Six sleep tracker applications have met inclusion/exclusion criteria and, thus, were included in the study. The two reviewers have evaluated the included apps for quality and subjectivity. Table 1 shows the average score between the two reviewers. Circadia track received the highest ranking for quality (3.6), followed by Sleeptic:Sleep Track & Smart AlarmClock (3.5), and Sleep CENTRAL (3.3). Circadia Track also had the highest subjectivity score (3.25), followed by Sleeptic:Sleep Track & Smart AlarmClock (2.88), and Zen Sleep Cycle Alarm Clock (2.75). The overall mean scores for quality and subjectivity were 3.3 and 2.66, respectively (Table 1). Table 2 shows mobile applications’ functionalities and description of apps objective quality, functionality, aesthetics, and information features are shown in Table 3.

**Table 1.** Mobile application rating scale scores.

App name	Quality reviewer 1	Quality reviewer 2	Average quality	Subjective reviewer 1	Subjective reviewer 2
**Airweave sleep analysis**	2.975	3.31	3.1425	2	2.5
**Circadia Track**	3.3525	3.78	3.56625	2.75	3.75
**JUKUSUI**	2.6625	3.49	3.07625	2	2.5
**Sleep CENTRAL**	3.2575	3.4	3.32875	2.75	2.5
**Sleeptic:Sleep Track & Smart AlarmClock**	3.475	3.6	3.5375	3	2.75
**Zen Sleep Cycle Alarm Clock**	3.34	3.2	3.27	3	2.5

**Table 2.** Mobile applications' functionalities.

App Name	Functionalities
Airweave sleep analysis	Sleep cycle measurement and graphing, smart alarm.
Circadia Track	Sleep diary, sleep metrics and sleep stages analysis, contactless sleep tracking.
Jukusui	Snoring logs, sleep log, music, smart alarm.
Sleep Central	Sleep, HR, RR*, and body movement recording, music, smart alarm.
Sleeptic:Sleep Track & Smart AlarmClock	Smart alarm, music, sleep recording and scoring, Google Fit integration.
Zen Sleep Cycle Alarm Clock	Smart alarm, sleep staging, dream log.

**Table 3.** Description of app quality rating (assessed by four domains of MARS).

App name	Engagement reviewer 1	Engagement reviewer 2	Mean engagement	Functionality reviewer 1	Functionality reviewer 2	Mean functionality	Aesthetics reviewer 1	Aesthetics reviewer 2	Mean aesthetics	Information reviewer 1	Information reviewer 2	Mean information
Airweave sleep analysis	3	3	3	3	4.25	3.625	3.3	3	3.15	2.6	3	2.8
Circadia Track	3.2	3.8	3.5	3.75	3.75	3.75	3.3	4	3.65	3.16	3.6	3.38
JUKUSUI	2.6	3.8	3.2	2.75	3.75	3.25	3	3.66	3.33	2.3	2.75	2.525
Sleep CENTRAL	3.2	3.6	3.4	4	3.75	3.875	3	3.33	3.315	2.83	3	2.915
Sleeptic:Sleep Track & Smart AlarmClock	3.6	3.8	3.7	4	4	4	4	4	4	2.3	2.66	2.48
Zen Sleep Cycle Alarm Clock	3.4	2.8	3.1	4	4	4	3.3	3.33	3.315	2.66	3	2.83

Assessment of perceptions about sleep behavioral change among users:

Table 4 shows characteristics of participants (n=30). Slightly under half of participants (46.7%, n=14) had prior experience with mobile health apps. Most were non-smokers (90%, n=27). Around 36.7% (n=11) did not exercise at all, while slightly over half (56.7%) exercised once to thrice a week. The majority of respondents were medically free (80%, n=24) and did not have any sleep disorders (73.3%, n=22), while only 10% (n=3) reported having insomnia and 16.7% (n=5) had bruxism. Sleep routine was also assessed for respondents (Table 5). Half of the study population did not have a bed or room partner, while the other half had one. The majority of participants (46.7%, n=14) reported going to bed between 10-12 p.m. During weekdays, most people (60%, n=18,) had to wake up before 9:00 a.m., while at weekends, only 53.3% woke-up before 9:00 a.m. Participant also achieved more actual sleep hours during weekends compared to weekdays. Subjects were asked to rate their sleep quality from “poor” to “very good”. Over half of participants (56.7%, n=17) rated their sleep quality as ‘good’.

**Table 4.** Characteristics of study participants.

Characteristics	n (%)
**Gender**	
Male	4 (13.3)
Female	26 (86.7)
**Marital status**	
Single	25 (83.3)
Married	5 (16.7)
**Highest education**	
High school	2 (6.7)
College student	28 (93.3)
**Occupation**	
Unemployed/housewife	5 (16.7)
Employed	6 (20)
Other	19 (63.3)
**Platform**	
Phone with Android operation system	18 (60)
iPhone	12 (40)
**Experience with mHealth app**	
Yes	14 (46.7)
No	16 (53.3)
**Smoking**	
Yes	3 (10)
No	27 (90)
**Exercise/week**	
I don’t exercise at all	11 (36.7)
Once/week	9 (30)
2 times or more/week	10 (33.3)

**Table 5.** Sleep routine and habits of study participants.

Characteristics	n (%)
**Snoring**	
Yes	3 (10)
No	18 (60)
I don’t know	9 (30)
**Bed partner or roommate**	
Yes	15 (50.0)
No	15 (50.0)
**Bedtime**	
Earlier than 10:00 p.m.	7 (23.4)
10:00 p.m. - 12:00 a.m.	14 (46.7)
Later than 12:00 a.m.	9 (30.0)
**Time to fall asleep**	
Less than 15 minutes	12 (40.0)
15-29 minutes	6 (20.0)
30 minutes or more	12 (40.0)
**Wake up time during weekdays**	
Earlier than 9:00 a.m.	27 (90.0)
9:00 a.m. or later	3 (10.0)
**Average sleep duration at weekdays**	
Less than 8 hours	20 (66.7)
8 hours or more	10 (33.3)
**Sleep duration on weekends**	
Less than 8 hours	12 (55.7)
8 hours or more	18 (45.3)
**Wake up time on weekends**	
Earlier than 9:00 a.m.	16 (53.3)
9:00 a.m. and later	14 (46.7)
**Self-rating of sleep quality**	
Very good	4 (13.3)
Good	17 (56.7)
Average	8 (26.7)
Poor	1 (3.3)
**Typical sleeping position**	
Back sleeping	3 (10)
Side sleeping	26 (86.7)
Stomach	1 (3.3)
Head elevated	0 (0)

**Figure 2.**
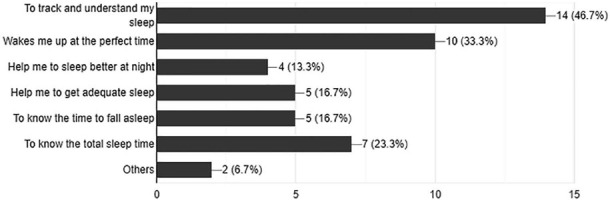
Reasons for using sleep tracker mobile applications.

Figure 2 shows the common reasons for using sleep apps among participants. Most participants reported using sleep trackers apps to track and gain understanding of their sleep (46.7%, n=14). Meanwhile, 33% (n=10) used such apps to wake up at the desirable time. Other targets for sleep apps use included improving sleep at night (13.3%, n=4), getting adequate sleep (16.7%, n=5), and gaining knowledge about the best sleep time (16.7%, n=5) and duration (23.3%, n=7).

After downloading and using sleep tracker apps included in the study, participants completed an app evaluation form based on the MARS tool (Table 6). About 36.6% of participants believed that sleep tracker apps are likely to increase awareness of the importance of addressing sleep patterns. A similar percent (33.3%) agreed that such apps are likely to increase knowledge/understanding of sleep hygiene. Most agreed that apps can affect the intent to change sleep hygiene habits (40%, n=12). Slightly below half agreed that sleep tracker apps are likely to encourage further help seeking for sleep hygiene when required. Finally, 33% of respondents agreed that sleep apps are likely to increase or decrease sleep hygiene. However, 40.0-53.3% of subjects of the study chose the option “neutral” for items of the MARS tool subscale. Table 7 shows app specific behavioral impact score. “Sleeptic: Sleep Track & Smart Alarm Clock” app had the highest app specific score based on the rating of five independent reviewers. However, “JUKUSUI” had the lowest app score.

**Table 6.** Participant perceptions to the behavioural changes subscale as adapted from MARS tool.

Item	n (%)
**Awareness:** this app is likely to increase awareness of the importance of addressing sleep patterns.	
1 (Strongly disagree)	2 (6.7)
2 (Disagree)	5 (16.7)
3 (Neutral)	12 (40)
4 (Agree)	10 (33.3)
5 (Strongly agree)	1 (3.3)
**Knowledge:** this app is likely to increase knowledge/understanding of sleep hygiene.	
1 (Strongly disagree)	2 (6.7)
2 (Disagree)	2 (6.7)
3 (Neutral)	16 (53.3)
4 (Agree)	9 (30)
5 (Strongly agree)	1 (3.3)
**Attitudes:** This app is likely to change attitudes toward improving sleep hygiene.	
1 (Strongly disagree)	2 (6.7)
2 (Disagree)	4 (13.3)
3 (Neutral)	13 (43.3)
4 (Agree)	11 (36.7)
5 (Strongly agree)	0 (0)
**Intention to change:** this app is likely to increase intentions/motivation to address sleep hygiene.	
1 (Strongly disagree)	2 (6.7)
2 (Disagree)	2 (6.7)
3 (Neutral)	14 (46.7)
4 (Agree)	12 (40)
5 (Strongly agree)	0 (0)
**Help seeking:** use of this app is likely to encourage further help seeking for sleep hygiene (if it is required).	
1 (Strongly disagree)	2 (6.7)
2 (Disagree)	2 (6.7)
3 (Neutral)	14 (46.7)
4 (Agree)	12 (40)
5 (Strongly agree)	0 (0)
**Perception about behaviour change:** use of this app is likely to increase/decrease sleep hygiene.	
1 (Strongly disagree)	2 (6.7)
2 (Disagree)	3 (10)
3 (Neutral)	15 (50)
4 (Agree)	10 (33.3)
5 (Strongly agree)	0 (0)

**Table 7.** App specific behavioural impact score.

	Strongly disagree	Disagree	Neutral	Agree	Strongly agree	Mean score	App score
**Sleeptic: Sleep Track & Smart AlarmClock**							
Awareness	0	0	2	2	1	3.8	3.50
Knowledge	0	0	2	2	1	3.8	
Attitudes	0	0	2	3	0	3.6	
Intention to change	0	0	3	2	0	3.4	
Help seeking	0	0	4	1	0	3.2	
Behaviour change	0	0	4	1	0	3.2	
**Airweave sleep analysis**							
Awareness	0	1	2	2	0	3.2	3.13
Knowledge	0	1	2	2	0	3.2	
Attitudes	0	1	3	1	0	3	
Intention to change	0	1	1	3	0	3.4	
Help seeking	0	1	3	1	0	3	
Behaviour change	0	1	3	1	0	3	
**Jukusui**							
Awareness	0	2	2	1	0	2.8	2.47
Knowledge	0	0	4	1	0	3.2	
Attitudes	2	1	1	1	0	2.2	
Intention to change	2	1	1	1	0	2.2	
Help seeking	2	1	1	1	0	2.2	
Behaviour change	2	1	1	1	0	2.2	
**Circadia Track**							
Awareness	2	1	1	1	0	2.2	3.27
Knowledge	2	0	1	2	0	2.6	
Attitudes	0	0	2	3	0	3.6	
Intention to change	0	0	2	3	0	3.6	
Help seeking	0	0	1	4	0	3.8	
Behaviour change	0	0	1	4	0	3.8	
**Sleep Central**							
Awareness	0	0	2	3	0	3.6	3.33
Knowledge	0	0	4	1	0	3.2	
Attitudes	0	2	2	1	0	2.8	
Intention to change	0	0	3	2	0	3.4	
Help seeking	0	0	1	4	0	3.8	
Behaviour change	0	1	2	2	0	3.2	
**Zen Sleep Cycle Alarm Clock**							
Awareness	0	1	3	1	0	3	3.16
Knowledge	0	1	3	1	0	3	
Attitudes	0	0	3	2	0	3.4	
Intention to change	0	0	4	1	0	3.2	
Help seeking	0	0	4	1	0	3.2	
Behaviour change	0	0	4	1	0	3.2	

## DISCUSSION

Over the past decade, mobile technology and smartphones have become an inspirational and integral part of modern living. Being widely used in the healthcare sector, mobile health and mHealth applications have started to establish their place and take part in healthcare^35,36^. In this study, we present a systematic evaluation of sleep tracker apps using the MARS tool. In addition, a MARS sub-scale tool was used for assessment of user perceptions about app-related behavioral changes in a sample of adults.

On a 5-point rating scale, the highest score achieved for quality was 3.6, compared to 3.25 in subjectivity, both achieved by the app “Circadia Track”. Meanwhile, the average MARS score for app quality among all applications was 3.3. This was similar to results from Choi et al. (2018)^22^, where the median overall MARS score reached 3.1. Choi et al. (2018)^22^ screened and evaluated 73 sleep health applications using the MARS tool and found that over half of the applications achieved a minimum acceptability score of 3.0. In general, such a mean of about 3 out of a 5 point on MARS scale indicate the need for more work to improve the validity, and benefts of these apps to users. Yet, apps included in this study offered multiple features such as tracking sleep stages, stage-specific alarms, heart rate and respiratory rate monitors, body movement monitors, and graph displays. Some of the apps, such as Sleep CENTRAL, also provided education on sleep hygiene improvement. In addition, the app Circadia Track offers analysis of sleep stages, circadian rhythm, and sleep metrics (e.g., sleep onset, time, and efficiency). Such differences in features could be suggested reasons for differences in results/scores among apps. It should also be considered that paid applications, which were excluded from this study, could offer additional functions, and have higher ratings.

To validate the algorithms used in calculating sleep parameters, multiple mobile applications for sleep tracking and management were tested in clinical studies^22^. Those apps were compared to standard sleep tests such as PSG, wrist actigraphy, and the Pittsburgh sleep quality index for validity and reliability in assessing sleep^24^. Parameters commonly assessed included total sleep time, sleep stages (wake, light sleep, deep sleep, rapid eye movement (REM), sleep latency, snoring events, sleep duration, and sleep efficiency). Correlation between the assessed apps and PSG varied according to study population (healthy vs. clinical) and the sleep parameter assessed. Good correlation with PSG was seen among parameter-based apps in detecting sleep stages, while accelerometer-based apps showed poor correlation with PSG sleep substages. For example, Bhat et al. (2015)^27^ assessed the accelerometer-based app “Sleep Time” in a sample of 20 healthy subject, who also underwent a full night sleep study using PSG. Despite having high sensitivity for detecting sleep and wake (89.9%), the app was not correlated with PSG sleep parameters. Meanwhile, results from the app “Sleep on Cue” were found to be significantly correlated to those of PSG regarding sleep onset latency (SOL)^37^. When compared to wrist actigraphy, sleep apps generally displayed accurate results in healthy populations. Meanwhile, a drop in accuracy is seen with both actigraphy and sleep trackers when assessing sleep-wake in clinical populations^24^. This was confirmed in a study where a noticeable decrease in reliability of measuring sleep parameters (e.g., total sleep time, waking after sleep onset, and sleep efficiency) was seen among patients with poorer sleep^1^.

Due to limitations in accuracy and reliability, sleep health applications may not be recommended in clinical population or as diagnostic tools^24,25,26,27,28,29,38^. Among participants of the current study, sleep apps were used for keeping track of sleep and increasing knowledge about the recommended sleep time and duration. About 13% also aimed to improve nighttime sleep using such apps. Therefore, they may prove useful as self-management methods for improving sleep hygiene and engaging patients in their therapy process. In fact, several studies have shown self-help and patient-centered interventions to be efficacious in improving sleep and sleep-related disorders^39,1^. For example, self-help cognitive behavioural therapy (CBT) has shown efficacy as a treatment for insomnia, and might be a useful alternative when face-to-face CBT is not possible^39^. In addition, sleep hygiene education has been proposed as a method for sleep management and improvement^40^.

Sleep hygiene (SH) refers to a set of practices and factors recommended for improving sleep that include exercise, stress management, limiting bedtime caffeine intake, establishing a consistent sleep routine, limiting daytime napping, among many others^41^. In two studies among university students in Kuwait and Hong Kong, sleep hygiene practice was shown to be associated with sleep quality^42,43^. Moreover, SH education is commonly indicated for managing sleep disorders, such as insomnia, with comparable or slightly lower efficacy than cognitive behavioural therapy^40^. In this study, we evaluated the behavioural changes associated with five sleep tracker apps as perceived by healthy adult participants. After using the apps, participants shared their assessment of app-related behavioural change aspects. Those included awareness, knowledge, attitudes, intention to change, help seeking, and perceived change of behaviour. Most participants believed the apps were helpful in increasing awareness and knowledge about sleep hygiene. Furthermore, they believed sleep trackers would lead to taking action in addressing sleep problems, including seeking help from professionals. Overall, participants agreed that the use of sleep trackers could affect sleep hygiene, either positively or negatively. Although data are limited about app-related behavioural change, a previous study found the app “Sleep Cycle” to be helpful in increasing awareness of sleep problems in children^44^. In our study, “Sleeptic: Sleep Track & Smart Alarm Clock” app was the most helpful app to increase awareness of the importance of addressing sleep patterns.

To our knowledge, this study is the first to assess perceptions about behavioural changes associated with sleep trackers from user’s perspective. This might prove helpful in highlighting the role of such apps in patient-centred self-management and sleep hygiene education. Additionally, an evaluation of sleep trackers apps available for mobile use was presented, with the aim of aiding physicians in choosing the most suitable app for patients. Current assessment was limited to applications available in English and requiring no subscription fees. Apps available on platforms other than Google Play Store or Apple App Store were also not included. Notably, in this study, we did not directly measure change in sleep hygiene. We rather assessed the perception of study participants about the effect of the use of the apps on user’s attitudes, knowledge, willingness to change, as well as its likelihood to change user’s behaviour. The term sleep hygiene was explained to the study participants as per its set criteria of practices^45^. Directly measuring the actual change in sleep hygiene, how those apps impact a person already has a good quality of sleep, or those who use the apps, but skip for some days will be the matter of our future work. Moreover, a comprehensive study that include measuring apps induced behavioural change among separate groups of ages, genders, patients with some typical diseases is warranted.

We believe that sleep apps may increase sleep quality by improving awareness about self-management, help seeking regarding sleep-related issues, and/or improvement of sleep hygiene. Another point of concern would be if any improvement in the sleep quality during the use of the apps is actually because of those apps themselves or because of patients’ thoughts about their sleep. To verify this, a separate comprehensive study is needed. Another possible limitation of this study is the use of e-mail lists to recruit subjects, which produced a relatively low response rate with majority of subjects being females. Thus, once again, a more comprehensive study is needed.

## CONCLUSION

Amid concerns regarding the limited validity of mobile sleep trackers and variation in results compared to golden standard PSG, other roles of such apps must be considered. We conclude that sleep trackers may be useful in improving user’s self-management, and increasing sleep hygiene awareness, knowledge, and behaviours. Thus, apps may present valuable tools for improving sleep quality. However, continuous audits and validation trials for available apps are vital to improve their quality. It is recommended to assess behavioral changes associated with sleep trackers in different populations, such as elders, and people with sleep disorders and major illnesses.
